# Mechanical Thrombectomy Versus Pharmacomechanical Thrombolysis in Arteriovenous Grafts: A Review

**DOI:** 10.7759/cureus.115102

**Published:** 2026-08-24

**Authors:** Ramanakumar Anam

**Affiliations:** 1 Nephrology, Louisiana State University Health Sciences Center Shreveport, Shreveport, USA

**Keywords:** angioplasty, avg, endovascular procedures, mechanical thrombectomy, pharmacomechanical thrombolysis

## Abstract

Arteriovenous grafts remain an essential hemodialysis access modality for patients with end-stage kidney disease, but their high incidence of thrombosis makes thrombotic occlusion a major cause of access dysfunction and permanent access loss. Mechanical thrombectomy and pharmacomechanical thrombolysis have consequently become the principal minimally invasive strategies for restoring graft function, largely replacing open surgical thrombectomy in contemporary practice. Although both endovascular approaches achieve high immediate technical success, neither reliably overcomes the poor long-term durability of thrombosed grafts largely because thrombosis is usually precipitated by an underlying anatomic stenosis that must be identified and treated. Available comparative evidence indicates that mechanical thrombectomy and pharmacomechanical thrombolysis provide broadly similar technical success and short-term patency, with no consistently demonstrated superiority of either declotting approach as a standalone strategy. However, durability after either salvage technique depends primarily on adjunctive treatment of the inciting venous anastomotic stenosis, where randomized data show that stent grafts and, in selected lesions, drug-coated balloons yield superior patency over plain balloon angioplasty. Methodological heterogeneity, small study populations, variable procedural techniques, and inconsistent definitions of patency limit the certainty and generalizability of all of these findings. This review compares the procedural mechanisms, efficacy, complication profiles, and predictors of patency associated with mechanical thrombectomy and pharmacomechanical thrombolysis, evaluates their implications for clinical decision-making, examines current guideline recommendations, and identifies priorities for more rigorous comparative research.

## Introduction and background

Functional vascular access is the lifeline of chronic hemodialysis, and the choice among arteriovenous fistulas, arteriovenous grafts, and central venous catheters has remained a major policy and clinical concern for more than three decades [1,2]. Although arteriovenous fistulas are generally preferred because of their superior long-term patency and lower infection risk, they are not feasible for all patients. Inadequate vasculature, previous failed access creation, and substantial comorbidity leave a significant proportion of patients dependent on arteriovenous grafts, which therefore remain the principal alternative to native fistulas [1,2]. This reliance is clinically important because grafts carry a distinct biological burden: stenosis due to neointimal hyperplasia - particularly at the venous anastomosis - drives recurrent thrombosis and infection, and serves as the principal substrate for progressive access failure [3,4]. The structural vulnerability of prosthetic conduits thus makes thrombosis a central therapeutic and health-care challenge rather than an episodic complication.

The most important clinical limitation of arteriovenous grafts is their pronounced susceptibility to thrombotic occlusion. Reported thrombosis rates range from 0.5 to 2.0 events per graft-year, with one cited estimate of 0.5 to 0.8 episodes per year - approximately two to three times higher than rates reported for arteriovenous fistulas - and access thrombosis accounts for an estimated 65% to 85% of permanent graft losses [2,5,6]. Each event commonly leads to missed dialysis sessions, hospital admission, and temporary central venous catheter placement; patients requiring a temporary catheter for urgent dialysis wait a median of four days for radiologic intervention, compared with two days among those who do not require catheter placement [6,7]. The available evidence suggests that technical success should not be equated with durable graft salvage: long-term patency after a single revascularization remains limited, with a median of less than 90 days in many series, and no individual declotting modality has demonstrated consistent superiority [5,8]. Contemporary pooled evidence reinforces this conclusion, as a 2024 systematic review and meta-analysis of six retrospective cohort studies involving 1,520 participants found no statistically significant difference between endovascular and surgical thrombectomy in one-year primary or secondary non-patency, with comparable technical failure rates [8,9]. Thus, the central clinical question is not simply which device achieves the highest immediate clot-removal rate, but whether the selected strategy effectively treats the underlying stenosis, minimizes procedural risk, and prolongs access usability, supporting an individualized approach based on lesion anatomy, thrombus burden, operator expertise, device availability, and the urgency of dialysis access restoration.

Three principal endovascular strategies have been evaluated for declotting thrombosed arteriovenous grafts. Pure mechanical thrombectomy physically fragments and removes thrombus using catheter-mounted devices without a thrombolytic agent, while pharmacomechanical thrombolysis combines catheter-directed delivery of a thrombolytic agent with mechanical maceration; both depend, for durable function, on adjunctive balloon angioplasty of the underlying culprit lesion with or without stent placement [10]. The third component is particularly important because declotting alone does not correct the stenosis that usually precipitates graft thrombosis and therefore cannot be expected to ensure durable access function. Within pharmacomechanical treatment, pulse-spray thrombolysis, mechanical clot maceration, and the lyse-and-wait technique represent established endovascular salvage strategies [11], and although their mechanisms differ, the clinical objective is the same: rapid restoration of flow followed by correction of the causative lesion. The earliest systematic evaluation of pharmacomechanical thrombolysis was the 1991 series of 121 thrombosed hemodialysis grafts treated with the Valji approach to catheter-directed clot lacing and pulse-spray urokinase: complete or near-complete thrombolysis was achieved in all 117 fully treated grafts, 93% of which remained patent at one day, with a mean pulse-spray lysis time of 46±21 minutes but one-year primary and secondary patency of only 26% and 51%, respectively [12,13]. Subsequent long-term follow-up and a comparative analysis of the original and current techniques confirmed that the current pulse-spray technique is as safe and effective as the original catheter-clot-lacing method, while iterative refinements have continued to shorten procedure times and reduce lytic exposure [12]. In contemporary practice, rotational thrombectomy devices such as the Cleaner XT are commonly used as the mechanical adjunct within a combined pharmacomechanical procedure, and a recent regional review observed that pharmacomechanical thrombolysis is a relatively simple, minimally invasive procedure that can facilitate rapid clot removal under local anaesthesia, with potentially reduced surgical exposure, cost, treatment time, and length of stay relative to surgery [11,14]. Accordingly, mechanical thrombectomy and pharmacomechanical thrombolysis have become the dominant endovascular strategies in contemporary practice and constitute the principal comparators in this review [5,6,8].

## Review

Methods

This narrative review was conducted to identify, critically appraise, and synthesize the comparative evidence on mechanical thrombectomy and pharmacomechanical thrombolysis for thrombosed arteriovenous grafts. PubMed/MEDLINE, Embase, the Cochrane Library, and Google Scholar were searched from database inception through July 2025. The literature search was closed in July 2025 to provide a clearly defined, reproducible endpoint that incorporates contemporary guideline updates, recent systematic reviews, and primary studies published through mid-2025, including the 2024 Cochrane review [9], the 2024 meta-analysis by Jatiman et al. [8], and the 2024 JAMA review by Lok and colleagues [15]. The search combined the core terms "arteriovenous graft," "thrombosis," "mechanical thrombectomy," and "pharmacomechanical thrombolysis" with related Medical Subject Headings and free-text variations, including "declotting," "AVG salvage," "pulse-spray thrombolysis," "lyse and wait," "AngioJet," "Arrow-Trerotola," "Cleaner XT," and "Indigo." Reference lists of included articles and recent systematic reviews and meta-analyses were also hand-searched to identify additional relevant sources.

Eligible studies comprised primary comparative cohort studies, randomized trials, systematic reviews and meta-analyses, and contemporary clinical practice guidelines addressing mechanical thrombectomy versus pharmacomechanical thrombolysis, or either endovascular approach versus surgical thrombectomy, in adults receiving haemodialysis for thrombosed arteriovenous grafts [16]. Direct comparative evidence was prioritized when available; indirect comparisons were used only to contextualize technical success, complications, and short- and long-term patency outcomes. Single-arm prospective series, technical notes, narrative reviews without primary data, and case reports involving fewer than ten patients were excluded, except when they provided unique technical detail relevant to the interpretation of a procedure or device.

Records were screened by title and abstract, and candidate full texts were assessed against the eligibility criteria above; reference-list hand-searching identified additional sources. The final evidence base comprised 45 sources, including primary comparative cohort studies and randomized trials, systematic reviews and meta-analyses, contemporary clinical practice guidelines, and a limited number of supporting technical and device-specific reports used for procedural context. Table 1 summarizes the principal comparative studies, their interventions, sample sizes, and patency outcomes.

Where sufficient evidence was available, effect estimates were reported as hazard ratios, odds ratios, or relative risks with 95% confidence intervals. When formal comparative estimates were unavailable, descriptive statistics were reproduced from the primary source and interpreted with attention to study design, sample size, follow-up duration, outcome definitions, and potential confounding. Particular emphasis was placed on the distinction between immediate technical success and durable access function, because successful clot removal does not necessarily correct the underlying stenosis or prevent recurrent thrombosis. Methodological strengths, limitations, and the clinical implications of the available evidence are summarized in the Evidence Quality and Limitations section. Because this review was narrative rather than systematic, it was intended to integrate heterogeneous comparative findings and clarify their clinical relevance rather than generate a pooled treatment-effect estimate; accordingly, no Preferred Reporting Items for Systematic Reviews and Meta-Analyses (PRISMA)-style flow diagram or PROSPERO registration is provided.

**Table 1:** Principal comparative studies of mechanical thrombectomy and pharmacomechanical thrombolysis in thrombosed arteriovenous grafts

Study	Comparison / intervention	Sample size	Key patency finding
Valji et al. [13]	Pharmacomechanical thrombolysis (pulse-spray urokinase)+angioplasty	121 grafts (117 fully treated)	93% patent at 1 day; 1-year primary 26%, secondary 51%
Thai retrospective cohort [17]	Endovascular therapy vs surgical thrombectomy	74 thrombosed grafts	Procedural success 92% vs 98%; 1-year primary patency 26% vs 33%; secondary 82.6% vs 56.3%
Randomized trial of mechanical device vs surgery [18,19]	Mechanical thrombectomy vs surgical thromboembolectomy	174 grafts (109 mechanical; 65 surgical)	No difference in immediate success or short- or long-term patency
Prospective randomized surgical vs endovascular trial [20]	Surgical thrombectomy/revision vs mechanical or pharmacologic thrombolysis+angioplasty	Not reported	Significant patency advantage for surgery (elastic venous anastomotic stenoses)
Pharmacomechanical vs surgical Thai cohort [21]	Pharmacomechanical thrombolysis vs surgical thrombectomy	Not reported	No differences between arms
AngioJet single-centre series [22,23]	Mechanical thrombectomy (rheolytic AngioJet)	92 procedures	Technical success 92.0%; clinical success 90.8%
Institutional endovascular salvage series [24,25]	Endovascular salvage (mechanical/pharmacomechanical)	328 procedures	6-month patency: 42.2%/46.7%/59.1%; 12-month patency: 23.4%/28.3%/41.8%; median access survival: 9.2 months
Meta-analysis of six cohort studies [8,9]	Endovascular vs surgical thrombectomy	1,520 participants	No significant difference in 1-year primary or secondary non-patency

Outcome definitions

To ensure consistency across the heterogeneous terminology used in the comparative literature, the following outcome definitions are applied throughout this review. Where cited studies used synonymous terms, equivalents are noted in brackets.

*Technical success*: Restoration of blood flow through the thrombosed arteriovenous graft at the end of the procedure, typically confirmed by completion fistulography. This is reported in this review as the proportion of procedures in which graft patency is re-established at procedure completion [6,8].

*Clinical success*: Successful use of the declotted graft for at least one haemodialysis session without access-related complication requiring unscheduled reintervention. This is reported as the proportion of procedures that translate into immediate dialysis usability [22,25].

*Primary patency*: Uninterrupted patency of the salvaged graft from the index intervention until the next access thrombosis or any repeat endovascular or surgical intervention performed to maintain or restore patency. This is reported as the cumulative proportion of grafts remaining patent without intervention over time [8,25,26].

*Primary-assisted patency*: Patency from the index intervention until access thrombosis, during which patency is maintained by prophylactic endovascular or surgical interventions performed before thrombosis occurs. This is reported in this review alongside primary patency to distinguish elective maintenance interventions from post-thrombosis salvage [25,26].

*Secondary patency*: Patency from the index intervention until final access abandonment, encompassing patency restored by interventions performed after a thrombotic event. This is reported as the cumulative proportion of grafts patent at the specified time point, with all repeat interventions counted toward outcome [8,25,26].

*Access-circuit survival:* The overall interval from creation of the access (graft or fistula) to abandonment of that vascular access site, encompassing all interventions performed on the same circuit. This is reported as a median time-to-abandonment rather than as a proportion at a fixed time point [15,25].

*"Non-patency"*: The cumulative probability that loss of patency has occurred by a given time point, calculated in time-to-event analysis as the complement of the cumulative patency probability, that is, 1−S(t), where S(t) denotes the Kaplan-Meier patency estimate at time t. This is reported in this review alongside the corresponding patency measures at specified time points; this convention allows direct comparison of event hazard across studies and is adopted in the 2024 meta-analysis by Jatiman et al. and the Cochrane review of interventions for thrombosed arteriovenous fistulas and grafts [8,9].

Technical approaches

Mechanical Thrombectomy

Mechanical thrombectomy employs catheter-mounted devices to macerate, fragment, or aspirate thrombus from the arteriovenous graft without the use of a thrombolytic agent. Contemporary device categories include rotational baskets and impellers, rheolytic saline-jet catheters, and aspiration thrombectomy catheters [6,27]. The procedure typically begins with sheath access into the thrombosed graft, followed by mechanical clot removal and a complete inflow-to-outflow angiogram to identify and treat the inciting stenosis [28]. Patency outcomes after mechanical extraction depend heavily on subsequent angioplasty of the underlying stenosis; at six months, primary patency is 19% after thrombectomy compared with 51% after elective angioplasty, and among grafts with no residual stenosis after intervention, median intervention-free patency is 2.5 months after thrombectomy versus 6.9 months after elective angioplasty [29,30].

A single-centre analysis of 92 mechanical thrombectomy procedures with the AngioJet system reported technical success in 92.6% of arteriovenous fistula cases and 92.0% of arteriovenous graft cases, with clinical success in 90.8% of arteriovenous graft cases, and significantly higher primary and primary-assisted patency rates in the arteriovenous fistula group; left-sided vascular access and female sex were independent predictors of failure for primary patency in arteriovenous grafts [22,23]. A larger institutional retrospective series of 328 endovascular salvage procedures performed over a decade reported a technical success rate of 87.8% and a clinical success rate of 75.9%, with primary, primary-assisted, and secondary patency at six months of 42.2%, 46.7%, and 59.1%, respectively, declining to 23.4%, 28.3%, and 41.8% at 12 months; median access circuit survival in that cohort was 9.2 months, the major complication rate was 5.2% including three procedure-related deaths, and use of AngioJet-based pharmacomechanical thrombectomy, native arteriovenous fistula, and shorter time from thrombosis to intervention independently predicted positive outcomes, with prior thrombectomy within three months and residual thrombus at procedure completion predicting poorer outcomes [24,25,31].

Pure mechanical thrombectomy is attractive in patients for whom thrombolytics are contraindicated - recently operated, recently stroke-affected, actively bleeding, or severely coagulopathic individuals [6,32]. The principal safety considerations include distal embolization of fragmented thrombus, hemolysis from rotating impellers, and, less commonly, vessel wall injury or dissection [6].

Pharmacomechanical Thrombolysis

Pharmacomechanical thrombolysis combines catheter-directed delivery of a thrombolytic agent - most commonly recombinant tissue plasminogen activator (rt-PA, alteplase) or, in earlier series, urokinase - with mechanical clot disruption and aspiration. The original "pulse-spray" technique uses high-pressure forceful injection through a multi-sidehole catheter to drive the thrombolytic agent radially into the clot while simultaneously macerating it, allowing lysis times on the order of less than one hour. The 1991 series of 121 thrombosed hemodialysis grafts treated with the Valji approach to pharmacomechanical thrombolysis reported complete or near-complete thrombolysis in 117 of 117 fully treated grafts, with 93% of grafts remaining patent at one day, mean pulse-spray lysis time of 46±21 minutes, and one-year primary and secondary patency rates of 26% and 51%, respectively; shorter intervals between thrombotic episodes were identified as the principal predictor of earlier subsequent failure [13]. Subsequent long-term follow-up from the same group showed comparable safety between the original and modified techniques, while iterative refinements have continued to shorten procedure times and reduce lytic exposure [12].

Pharmacomechanical thrombolysis is conceptually attractive because the thrombolytic agent addresses residual thrombus not reached by mechanical manipulation, while the mechanical component shortens overall procedure time and reduces the cumulative dose of thrombolytic required. Specific bleeding risks related to thrombolytic administration - including puncture-site hemorrhage, gastrointestinal bleeding, and remote bleeding - distinguish pharmacomechanical from purely mechanical approaches. In contemporary practice, rotational thrombectomy devices such as the Cleaner XT are commonly used as the mechanical adjunct within a combined pharmacomechanical procedure [11,14].

Comparative efficacy

Technical and Clinical Success

Across contemporary comparative studies, mechanical thrombectomy and pharmacomechanical thrombolysis achieve broadly comparable immediate success rates, with technical success typically reported in the 80%-95% range depending on access type, case mix, and operator experience [8,19,23,25,27]. A retrospective Thai cohort of 74 thrombosed dialysis grafts reported procedural success rates of 92% with endovascular therapy and 98% with open surgical thrombectomy, with no significant difference between the groups, indicating comparable procedural success between the two technique families [17].

Patency Outcomes

Long-term patency after a single revascularization remains limited regardless of the endovascular technique. Moreover, a clear separation in patency emerges between elective angioplasty and thrombectomy cohorts: at six months, the primary patency rate is 19% after thrombectomy versus 51% after elective angioplasty, while median intervention-free patency among grafts with no residual stenosis is 2.5 months after thrombectomy versus 6.9 months after elective angioplasty. Taken together, these findings have framed the contemporary argument that proactive detection and treatment of graft stenosis is preferable to reactive declotting after thrombosis has occurred [29,30].

In the Thai comparative cohort, one-year primary patency was 26% after endovascular therapy and 33% after surgical thrombectomy, whereas secondary patency was 82.6% and 56.3%, respectively [17,31]. Similarly, an institutional series of 328 endovascular salvage procedures reported a median access-circuit survival of 9.2 months, with progressive decline in primary, primary-assisted, and secondary patency over time [25,33].

A prospective randomized comparison of endovascular and surgical management of thrombosed dialysis access grafts, described as one of the largest such trials in the literature [5], found a statistically significant advantage in patency rate with surgical thrombectomy and revision compared with mechanical or pharmacologic thrombolysis and angioplasty, which the authors attributed to the limited capacity of balloon angioplasty to address the predominantly elastic stenoses that typically occur at the venous anastomosis - stenoses with limited plaque to crack and substantial recoil [34].

Specific Risks

Both mechanical thrombectomy and pharmacomechanical thrombolysis carry the specific risks of symptomatic pulmonary embolism, arterial embolism through paradoxical or direct embolization, cerebral embolism, perigraft hematoma or hemorrhage, and vein rupture, although major complications of percutaneous treatment are relatively uncommon in aggregate [6,32]. Percutaneous declotting should be avoided in clotted mega-fistulas, in which the large volume of clot can cause symptomatic pulmonary embolism even in patients with normal cardiopulmonary reserve; in patients with a known right-to-left shunt, in whom embolized clot can enter the central arterial circulation and cause stroke or other systemic complications; and in patients with access infection, hemodynamic instability, hyperkalemia, severe coagulopathy, recent access creation within four to six weeks, or severe cardiopulmonary reserve impairment [6]. In the early Valji series of pharmacomechanical thrombolysis, gastrointestinal bleeding requiring transfusion was observed in less than 1% of patients and minor complications in 3% [13].

Predictors of outcome

Timing of Intervention

Earlier intervention after thrombosis is consistently associated with improved technical success and improved patency. Contemporary evidence identifies that the optimal time of percutaneous pharmacomechanical thrombolysis for treating thrombosed arteriovenous grafts is within 48 hours of the onset of thrombosis for enhancing procedural success and patency rates [35,36]. Lower time from thrombosis to intervention has similarly been independently associated with positive outcomes in decade-long institutional experience [25]. Conversely, the European clinical practice guideline group has argued that acute thrombosis is associated with vessel wall inflammation and endothelial injury, and that such early active inflammation may itself be prothrombotic; accordingly, although immediate intervention is intuitively attractive, the existing observational data are limited and at high risk of bias, and an adequately powered prospective trial assessing standardized outcome measures across increasing time-to-intervention analysed as a continuous variable is needed [3,16].

Prior Thrombosis History

A history of recurrent thrombosis is one of the most powerful negative predictors of durable patency. The original Valji experience identified shorter intervals between thrombotic events as the principal predictor of earlier subsequent graft failure [13,24,37]. In a decade-long retrospective endovascular salvage cohort, prior thrombectomy within three months and residual thrombus at procedure completion were independently associated with poorer outcomes, including loss of primary, primary-assisted, and secondary patency [25].

Underlying Stenosis

The presence and severity of an underlying anatomic stenosis - almost always at the venous anastomosis or in the draining vein - is the dominant determinant of post-thrombectomy durability. Stenosis due to intimal hyperplasia at outflow veins is the principal biological basis of recurrent dialysis access failure [3,38]. The Society of Interventional Radiology quality improvement guidelines suggest that surgical revision be considered when angioplasty is required more than two times within three months or when thrombosis occurs more than two times within one month, and the Kidney Disease Outcomes Quality Initiative (KDOQI) guidelines similarly suggest that surgical revision should be considered under analogous repetitive-intervention criteria [3,26]. Inadequate treatment of the inciting stenosis at the time of thrombectomy contributes to early re-thrombosis regardless of which declotting technique is employed [39,40].

Access Type

Patency after thrombectomy differs meaningfully between arteriovenous fistulas and arteriovenous grafts. Native fistulas retain higher long-term patency after salvage than prosthetic grafts [23,25], and arteriovenous fistula was an independent predictor of positive outcome in decade-long institutional experience [23,25]. The presence of prosthetic material within the conduit is associated with more rapid intimal hyperplasia progression, earlier recurrence of stenosis, and a higher rate of rethrombosis [4,41], and these biologically determined disadvantages of grafts persist across both endovascular and surgical salvage approaches.

Current guidelines and recommendations

The European clinical practice guidelines on peri- and postoperative care of arteriovenous fistulas and grafts for haemodialysis in adults, summarizing the underlying evidence in 2019, concluded that the choice between surgical and endovascular interventions for arteriovenous (AV) access thrombosis should be defined by the condition of the patient and their vascular access, as well as local expertise, as there is no evidence that one approach improves outcomes more than any other (Grade 2B) [16,42]. The guideline group emphasized that thrombosis is one of the most frequent causes of access failure and that successful declotting can save the access from permanent failure, while also noting that the intuitive view that AV access thrombosis is an emergency necessitating immediate intervention is supported only by very sparse evidence - an adequately powered prospective observational trial assessing standardized outcome measures across increasing time-to-intervention is identified as a key research priority [16]. Comparator guidelines cited in the same European document recommend: for the Canadian Society of Nephrology, correcting thrombosis of an AV graft with pharmacomechanical or mechanical thrombolysis or surgical thrombectomy; for the European Society for Vascular Surgery, that surgery or endovascular methods should be considered for treatment of late thrombosis of vascular accesses depending on the centre's expertise; for the European Society for Vascular Surgery, that treatment of vascular access thrombosis should always include perioperative diagnosis and treatment of any associated stenosis; and for the Spanish Multidisciplinary Group on Vascular Access (GEMAV) group, that native AV fistulae with thrombosis secondary to juxta-anastomotic stenosis be treated surgically where central venous catheter placement is not required, and that thromboses not associated with juxta-anastomotic stenosis be restored by surgical treatment or by endovascular therapy using mechanical thrombectomy or aspiration devices where necessary [16].

The KDOQI Clinical Practice Guideline for Vascular Access acknowledges that both endovascular and open surgical approaches for treating thrombosed arteriovenous grafts are acceptable, that the choice is determined by local practice and expertise, the underlying anatomic substrate, and patient preference, and that open surgical and endovascular approaches should not be viewed as competing but rather as complementary, with hybrid approaches combining both techniques offering optimal results in selected patients [26,43]. Contemporary review adds that, regardless of declotting technique, endovascular or surgical correction of underlying stenotic lesions remains essential and typically involves balloon angioplasty with or without drug-coated balloons or stenting [15,34,44].

Evidence quality and limitations

The most important limitation of the comparative literature on mechanical thrombectomy versus pharmacomechanical thrombolysis for thrombosed arteriovenous grafts is the overall quality and consistency of available evidence. A contemporary Cochrane systematic review of interventions for thrombosed hemodialysis AV fistulas and grafts concluded that there is insufficient evidence to suggest that a specific intervention is better than another for outcomes including failure, primary patency at 30 days, technical success, and adverse events, and that most included studies were at high risk of bias due to poor study design, small sample sizes, and industry involvement [8,9]. Beyond aggregate quality concerns, several specific limitations constrain the interpretation of individual studies. Patency definitions are inconsistent across the literature, with primary, primary-assisted, and secondary patency each used variably, and follow-up durations rarely extending beyond 12 to 24 months. Most comparative cohorts are retrospective and small, typically in the range of 20-200 patients per arm. Selection bias is substantial because patients with shorter expected survival, fewer alternative access sites, or higher surgical risk are disproportionately referred to endovascular salvage. The endovascular arm of many pooled analyses bundles mechanical thrombectomy and pharmacomechanical thrombolysis together, complicating any attempt to differentiate outcomes between the two strategies. Finally, iterative device evolution means that contemporary mechanical devices bear little resemblance to the early mechanical devices evaluated historically, so older comparative cohorts may not accurately reflect present-day practice.

Hybrid procedures that combine surgical thrombectomy with high-pressure balloon angioplasty of the venous anastomotic stenosis have been proposed as a third option [43], with single-centre experience describing such hybrid salvage as a highly successful and safe procedure with acceptable short-term primary patency, while emphasizing that continuous graft monitoring and surveillance are needed to improve assisted primary and secondary patency rates. Other single-centre comparative cohort evidence suggests that, despite being safe and minimally invasive, hybrid treatment for AV graft thrombosis associated with venous anastomotic stenosis achieves competitive results compared with open surgery only after a second iterative procedure, and that the technique should be reserved for difficult surgical-approach stenoses owing to associated costs [45].

Clinical implications and future directions

Based on the synthesis of contemporary evidence, several clinical implications emerge for the management of thrombosed arteriovenous grafts. First, mechanical thrombectomy and pharmacomechanical thrombolysis offer broadly equivalent short-term efficacy: both approaches consistently achieve high technical and clinical success rates, with no reliable separation between the two in immediate procedural outcomes or in short-term patency. Either technique is therefore a reasonable first-line endovascular option for AVG declotting when endovascular salvage is indicated, and the choice between them should be guided by operator experience, available equipment, bleeding risk, and time since thrombosis rather than by an anticipated superiority of one technique over the other [8,14,19,21].

Second, in the absence of clearly demonstrable superiority, the choice between mechanical thrombectomy and pharmacomechanical thrombolysis should be guided primarily by institutional and patient-level factors. Centres with extensive experience and contemporary equipment for a particular device family will typically achieve the best outcomes with that family. Mechanical thrombectomy is preferred when thrombolytic agents are contraindicated (recent surgery, active bleeding, recent stroke, severe coagulopathy, high risk of intracranial hemorrhage), whereas pharmacomechanical thrombolysis is preferred when thrombus burden is large, when a low-dose lytic regimen can shorten procedure time relative to mechanical-only maceration, and when the operator has experience with catheter-directed pulse-spray or equivalent techniques [6,12,32].

Third, surgical thrombectomy retains a specific role for selected patients. For those with longer life expectancy, limited alternative access options, and anatomy favouring surgical revision, surgical thrombectomy combined with proximalization of the venous anastomosis appears to offer superior long-term patency beyond 90 days in some series, particularly when the predominant stenotic lesion is elastic and recoil-prone (the predominant morphology at the venous anastomosis) [8,15,16,20]. However, contemporary pooled endovascular data have not consistently demonstrated a patency disadvantage for endovascular therapy, and the surgical-versus-endovascular choice is generally individualized.

Fourth, the relationship between surveillance and graft longevity requires precise framing. Routine clinical monitoring - physical examination of the access at each dialysis session - is recommended standard care and should be distinguished from technical surveillance with pre-emptive angioplasty. For arteriovenous grafts specifically, the European clinical practice guideline does not recommend routine duplex ultrasound surveillance combined with pre-emptive balloon angioplasty, concluding that this strategy has not been shown to prevent thrombosis or improve graft functionality, whereas the same guideline suggests surveillance and pre-emptive angioplasty for fistulas [16]. The 2019 KDOQI update likewise emphasizes that stenosis detected by surveillance in the absence of clinical indicators should not be treated: for grafts, available data did not demonstrate improved patency with surveillance and subsequent pre-emptive intervention compared with routine clinical examination, and several studies previously rated high-quality were found to be underpowered, contributing to the change in recommendations [46]. Randomized evidence is concordant: among 112 patients, monthly access-flow surveillance increased stenosis detection and intervention rates (51 vs. 31 interventions) without reducing graft thrombosis (0.51 vs. 0.41 per patient-year) or prolonging time to graft loss (P=0.890) [47] and no non-invasive test currently distinguishes reliably between stenosed grafts that will and will not thrombose [30]. Pre-emptive angioplasty should therefore be reserved for haemodynamically significant stenosis accompanied by clinical indicators, while clinical monitoring anchors routine access care [16,46]. Correction of the underlying stenotic lesion during the same procedure as declotting, by contrast, is mandatory for durable salvage and must not be omitted simply because flow has been restored [16].

Fifth, endovascular salvage should be pursued as early as feasible - optimally within 24 to 48 hours of thrombosis onset - to maximize procedural success and patency [35,36], although the supporting evidence is limited to observational data and a well-powered prospective assessment of time-to-intervention remains a research priority [16].

Sixth, hybrid procedures combining surgical thrombectomy with adjunctive endovascular balloon angioplasty, with or without stenting, may optimize outcomes in selected patients with complex anatomy or juxta-anastomotic stenoses not amenable to standard angioplasty, although the available evidence remains limited to single-centre series [43,45].

Future research priorities include randomized head-to-head comparisons of mechanical thrombectomy versus pharmacomechanical thrombolysis using standardized patency definitions and event adjudication, longer-term follow-up extending reliably beyond two years, dedicated study of newer-generation mechanical devices and conventional pharmacomechanical combinations against contemporary reference standards, cost-effectiveness analyses integrating device cost, lytic cost, hospitalization duration, and repeat interventions, and patient-centred outcomes research incorporating quality of life, treatment burden, and patient preferences. Integration of these data into future iterations of the KDOQI and European guidelines will sharpen the basis on which clinicians select between these closely competing strategies.

## Conclusions

Mechanical thrombectomy and pharmacomechanical thrombolysis are both effective minimally invasive options for thrombosed arteriovenous hemodialysis grafts. Both approaches achieve high immediate technical and clinical success rates and broadly comparable short-term patency outcomes, and both share the constraint that long-term durability is limited primarily by the underlying anatomic stenosis rather than by the choice of declotting technique. Contemporary pooled estimates indicate no statistically significant difference between endovascular and surgical thrombectomy in one-year primary or secondary patency, although the most informative prospective randomized comparison and certain earlier pooled syntheses have reported a modest long-term advantage for surgical thrombectomy, possibly attributable to more durable treatment of elastic venous anastomotic stenoses. The choice between mechanical thrombectomy and pharmacomechanical thrombolysis should be individualized on the basis of operator expertise, anatomic suitability, bleeding risk, time since thrombotic event, and availability of devices and thrombolytic agents. Adjunctive balloon angioplasty of the underlying stenosis, ideally during the same procedure, is mandatory for durable salvage. Priorities for future research include randomized comparative trials, standardization of patency reporting, and integration of patient-centred outcomes.
